# Involvement of Oxidative Stress-Related Inflammatory Mediators in the Pathogenesis and Treatment Response of Macular Edema Secondary to Branch Retinal Vein Occlusion

**DOI:** 10.3390/antiox15050607

**Published:** 2026-05-11

**Authors:** Takuto Yamamoto, Hidetaka Noma, Tatsuya Mimura, Shotaro Sasaki, Taro Otawa, Kanako Yasuda, Masahiko Shimura

**Affiliations:** 1 Department of Ophthalmology, Hachioji Medical Center, Tokyo Medical University, 1163, Tatemachi, Hachioji 193-0998, Japan; takuto2584@gmail.com (T.Y.); hanshinchelsea@yahoo.co.jp (S.S.); kana6723@yahoo.co.jp (K.Y.); masahiko@v101.vaio.ne.jp (M.S.); 2 Department of Ophthalmology, Tokyo Medical University Ibaraki Medical Center, 3-20-1 Chuo, Amimachi, Inashiki-gun 300-0395, Japan; otawa.positive@gmail.com; 3 Department of Ophthalmology, Tsurumi University School of Dental Medicine, 2-1-3 Tsurumi, Tsurumi-ku, Yokohama 230-0063, Japan; mimurat-tky@umin.ac.jp

**Keywords:** oxidative stress, species, macular edema, branch retinal vein occlusion, anti-VEGF therapy, FMS-related tyrosine kinase 3 ligand, CXCL-16, endocan-1

## Abstract

*Background*: Branch retinal vein occlusion (BRVO) represents a segmental retinal ischemic disorder characterized by localized oxidative–inflammatory activation. While redox-driven cytokine responses have been described in central retinal vein occlusion, their role in BRVO-specific macular edema and treatment responsiveness remains unclear. This study investigated whether novel redox-related inflammatory mediators in the aqueous humor are associated with disease severity and structural response to anti-vascular endothelial growth factor (VEGF) therapy in BRVO. *Methods*: Aqueous humor samples were collected from 30 treatment-naïve patients with BRVO and 19 control patients. Levels of VEGF and the novel redox-related inflammatory factors FMS-related tyrosine kinase 3 ligand (Flt-3L), fractalkine, CXCL-16, and endocan-1 were measured by suspension array, and the severity of macular edema was evaluated by measuring central macular thickness and neurosensory retinal thickness (TNeuro) by spectral-domain optical coherence tomography. Therapeutic response was assessed one month after intravitreal ranibizumab injection (IRI). *Results*: Aqueous levels of VEGF, Flt-3L, and endocan-1 were significantly higher in the BRVO group, and levels of Flt-3L, CXCL-16, and endocan-1—markers associated with oxidative endothelial damage and leukocyte recruitment—correlated significantly with each other and with aqueous flare values. Notably, baseline Flt-3L levels significantly correlated with the reduction in TNeuro, suggesting that this redox-sensitive signaling molecule is a potential biomarker for treatment sensitivity. *Conclusions*: These findings suggest that novel inflammatory factors, potentially driven by oxidative-nitrosative stress, play a pivotal role in the pathophysiology of BRVO. Baseline Flt-3L may serve as a predictive biomarker for structural responsiveness to anti-VEGF therapy in BRVO, suggesting that oxidative–inflammatory signaling contributes not only to disease severity but also to therapeutic heterogeneity.

## 1. Introduction

Branch retinal vein occlusion (BRVO) results from vascular blockage commonly linked to atherosclerotic disease, initiating a cascade of mechanical, ischemic, and inflammatory responses [1]. Macular edema frequently complicates BRVO and remains the principal cause of vision loss in affected patients. The development of macular edema is driven by multiple angiogenic and inflammatory cytokines, with vascular endothelial growth factor (VEGF) playing a central role [1,2,3].

Emerging data indicate that BRVO pathophysiology extends beyond simple vascular obstruction and involves a marked oxidative–inflammatory response [4,5]. Hypoxia following venous occlusion promotes overproduction of reactive oxygen species (ROS) [6], which activate redox-sensitive transcriptional regulators such as nuclear factor-kappa B [7]. Activation of these pathways enhances expression of pro-angiogenic and pro-inflammatory mediators, including VEGF [8]. Although intravitreal anti-VEGF therapy is the current standard for treating BRVO-related macular edema [9,10], a substantial number of patients show inadequate response or experience recurrence [11,12]. These clinical limitations imply that additional redox-regulated mechanisms—and possibly factors like FMS-related tyrosine kinase 3 ligand (Flt-3L), fractalkine, CXCL16, and endocan-1—may play important roles in disease persistence and severity.

While inflammation’s contribution to BRVO is well recognized [13], dependable biomarkers that predict disease activity or therapeutic response have not been established. Because both VEGF-driven and inflammatory pathways are implicated, uncovering new intraocular mediators has clinical importance. Accordingly, this study measured aqueous humor concentrations of several novel inflammatory molecules in patients with BRVO-associated macular edema and assessed their relationships with edema severity and their potential utility as prognostic biomarkers for outcomes following anti-VEGF treatment.

## 2. Materials and Methods

### 2.1. Participants and Clinical Data

Thirty untreated patients with BRVO-associated macular edema and 19 cataract patients without retinal pathology were consecutively enrolled at Tokyo Medical University Hachioji Medical Center (Tokyo, Japan). Cataract cases served as controls because aqueous humor could be safely obtained during surgery. BRVO patients received an initial intravitreal ranibizumab injection (IRI) (Lucentis; 0.5 mg). Patients were divided into two groups based on the site of retinal vein occlusion: the major BRVO group, characterized by occlusion of a major branch retinal vein, and the macular BRVO group, characterized by occlusion of a macular venule.

Eligibility criteria included acute BRVO with foveal involvement, best-corrected visual acuity (BCVA) better than 25/30 (LogMAR), and central retinal thickness (CMT) exceeding 300 μm measured by SD-optical coherence tomography (OCT) (Spectralis; Heidelberg Engineering, Heidelberg, Germany). Patients unable to maintain fixation were excluded. Additional exclusions comprised ischemic BRVO confirmed by fluorescein angiography (≥5 nonperfusion areas) [14], macular ischemia, diabetic retinopathy, advanced cataract, other ocular disorders, prior anti-VEGF therapy, and previous ocular interventions.

Clinical data including BCVA, aqueous flare, and OCT parameters were extracted from medical records (see [1]). The study was approved by the institutional ethics committee and conducted in accordance with the Declaration of Helsinki, with written informed consent obtained from all participants.

### 2.2. Aqueous Humor Sampling

Under topical anesthesia, approximately 0.1 mL of aqueous humor was aspirated through the limbus using a 30 G needle in BRVO patients, while control samples were collected during cataract surgery. All specimens were stored at −80 °C until analysis.

### 2.3. OCT Evaluation of Macular Edema

**Figure 1:**
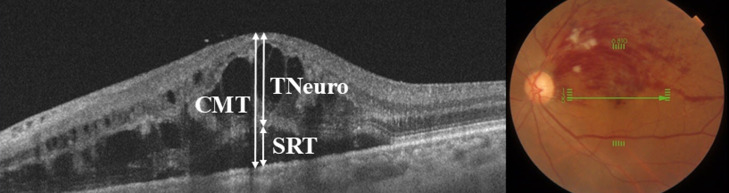
Measurement of central macular thickness (CMT), subfoveal neurosensory retinal thickness (TNeuro), and serous retinal thickness (SRT) using optical coherence tomography (OCT). The representative OCT image demonstrates the manual segmentation of CMT, TNeuro, and SRT.

SD-OCT was performed one week prior to treatment to quantify CMT, subfoveal neurosensory retinal thickness (TNeuro), and serous retinal thickness (SRT) [3,15]. High-reproducibility 6 mm horizontal and vertical scans centered on the fovea were obtained by a single experienced examiner. The severity of macular edema was classified based on CMT, TNeuro, and SRT, which were defined as follows (Figure 1) [3]: (1) CMT was measured as the distance between the inner limiting membrane and the basal membrane of the retinal pigment epithelium; (2) TNeuro was defined as the thickness of the subfoveal neurosensory retina; and (3) SRT represented the subfoveal serous retinal detachment height. All measurements were conducted using the OCT software (HEYEX 1.10)’s built-in calipers by two retinal specialists blinded to the patients’ BCVA and cytokine levels.

### 2.4. Intravitreal Injection and Outcome Assessment

Ranibizumab was delivered through the pars plana using a 30 G needle placed 3.5 mm posterior to the limbus in the superotemporal quadrant, followed by topical antibiotics for three days. Four weeks later, BCVA, aqueous flare, and OCT measurements were repeated. Treatment response was expressed as the percentage reduction in CMT and TNeuro: %ΔX = (Xpre − Xpost)/Xpre × 100.

### 2.5. Cytokine Analysis

Aqueous concentrations of VEGF, Flt-3L, fractalkine, CXCL-16, and endocan-1 were quantified using a Luminex-based multiplex assay (xMAP; Luminex Corp., Austin, TX, USA) by using the appropriate Milliplex kit, i.e., the Human Cytokine/Chemokine kit (HCYTA-60K; Merck Millipore, Billerica, MA, USA) for VEGF, Flt-3L, and fractalkine and the Human Cardiovascular Disease kit (HCVD1MAG-67K; Merck Millipore, Billerica, MA, USA) for CXCL-16 and endocan-1 with Milliplex kits as previously described [16,17]. Median fluorescence values were converted to concentrations using a five-parameter logistic model.

### 2.6. Statistical Methods

Results are reported as mean ± SD. Group comparisons were performed using Student’s *t* test or Mann–Whitney U test as appropriate. Correlations were assessed with Pearson or Spearman analyses. Statistical significance was defined as *p* < 0.05. All analyses were conducted with SAS software version 9.4.

## 3. Results

### 3.1. Patient Characteristics

The mean (SD) age was 67.7 (11.0) years in the BRVO group (14 men, 16 women) and 67.8 (4.7) years in the control group (9 men, 10 women). No significant differences were observed between the two groups in terms of age (*p* = 0.970) or sex (*p* = 0.962). Hypertension was significantly more prevalent in the BRVO group than in the control group (73.3% [22/30] vs. 10.5% [2/19]; *p* < 0.001), as was hyperlipidemia (53.3% [16/30] vs. 15.8% [3/19]; *p* = 0.009). In the BRVO group, the mean (SD) pre-treatment symptom duration was 42.1 (36.2) days (range, 7–144 days).

Within the BRVO group, 18 patients had major BRVO (7 men, 11 women; mean age, 67.5 [10.0] years) and 12 patients had macular BRVO (7 men, 5 women; mean age, 68.1 [12.8] years). There were no significant differences between the major and macular BRVO subgroups regarding age (*p* = 0.887), sex (*p* = 0.296), hypertension (72.2% vs. 75.0%; *p* = 0.866), hyperlipidemia (55.5% vs. 41.7%; *p* = 0.765), or pre-treatment symptom duration (50.0 [42.0] vs. 30.3 [21.8] days; *p* = 0.147).

### 3.2. Changes in BCVA, SD-OCT Findings, and Aqueous Flare Values After IRI

Four weeks after IRI, BCVA was significantly improved (pre-treatment value, 0.36 ± 0.28 logMAR; post-treatment value, 0.17 ± 0.32 logMAR; *p* < 0.001). In addition, a significant decrease was measured in CMT (pre-treatment value, 443 ± 124 μm; post-treatment value, 251 ± 64.2 μm; *p* < 0.001); TNeuro (pre-treatment value, 527 ± 137 μm; post-treatment value, 265 ± 76.9 μm; *p* < 0.001); SRT (pre-treatment value, 84.8 ± 84.2 μm; post-treatment value, 13.5 ± 32.6 μm; *p* < 0.001); and aqueous flare (pre-treatment value, 8.21 ± 3.30 photon counts/ms; post-treatment value, 5.81 ± 2.50 photon counts/ms; *p* < 0.001).

### 3.3. Comparison of Cytokine Levels Between the BRVO and Control Groups

VEGF and all novel inflammatory factors were detectable in the aqueous humor samples (lowest detectable concentrations: VEGF, 26.3 pg/mL; Flt-3L, 5.4 pg/mL; fractalkine, 22.7 pg/mL; CXCL-16, 13.2 pg/mL; and endo-can-1, 11.5 pg/mL). Concentrations of VEGF, Flt-3L, and endocan-1 were significantly higher immediately before IRI in the BRVO group than at the start of cataract surgery in the control group (Table 1), but those of fractalkine and CXCL-16 were not (Table 1).

### 3.4. Relationship Between Cytokine Levels and Baseline SD-OCT Findings and Aqueous Flare Values

**Figure 2:**
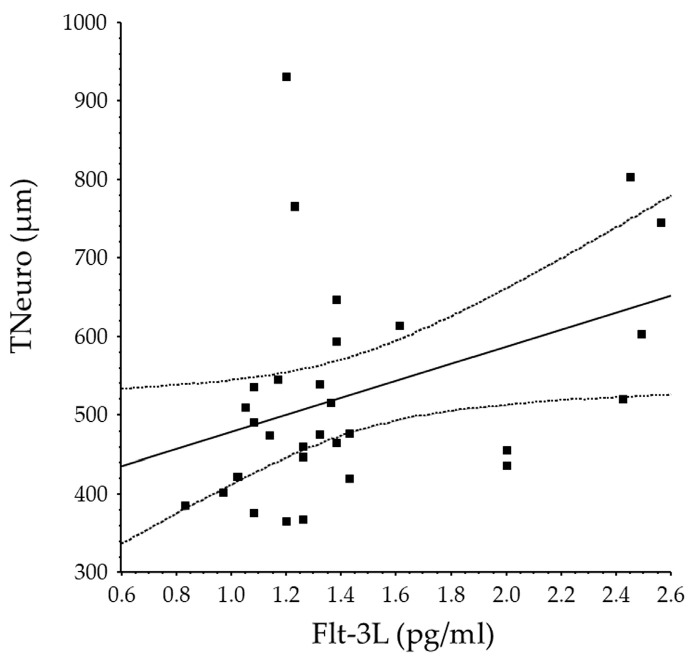
Correlation between baseline aqueous Flt-3L levels and baseline TNeuro. The baseline aqueous humor level of Flt-3L was significantly correlated with baseline TNeuro (*r* = 0.39, *p* = 0.031).

In the BRVO group, significant correlations were found between CMT and VEGF; TNeuro and Flt-3L (Figure 2); and the aqueous flare value and VEGF, Flt-3L, CXCL-16, and endocan-1 (Table 2).

### 3.5. Correlations Among the Cytokines in the BRVO Group

In the BRVO group, significant correlations were found at baseline between VEGF and endocan-1; between Flt-3L and fractalkine, CXCL-16, and endocan-1; between fractalkine and CXCL-16; and between CXCL-16 and endocan-1 (Table 3).

### 3.6. Association of Cytokines with Clinical Parameter Changes

**Figure 3:**
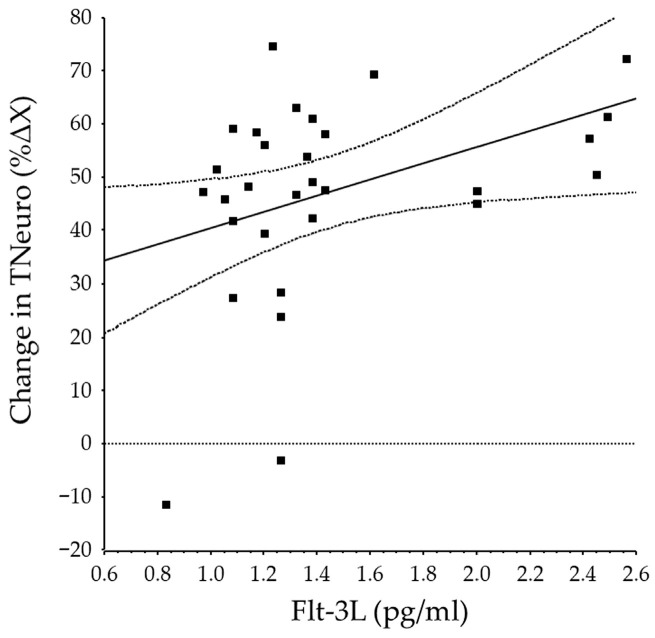
Correlation between baseline aqueous Flt-3L levels and changes in TNeuro. The baseline aqueous humor level of Flt-3L was significantly correlated with the change in TNeuro (*r* = 0.39, *p* = 0.032).

In the BRVO group, baseline aqueous humor levels of VEGF, Flt-3L, fractalkine, CXCL16, and endocan-1 were not significantly correlated with improvement in BCVA or change in aqueous flare value (Table 4). The baseline aqueous humor level of Flt-3L was significantly correlated with changes in CMT and TNeuro (Figure 3), but the baseline aqueous humor levels of VEGF, fractalkine, CXCL-16, and endocan-1 were not (Table 4).

## 4. Discussion

BRVO is a segmental retinal ischemic disorder characterized by localized oxidative and inflammatory activation [18]. Although redox-driven cytokine responses have been well-documented in central retinal vein occlusion (CRVO), their specific roles in BRVO-associated macular edema and its responsiveness to treatment remain poorly understood. This study aimed to elucidate whether novel redox-related inflammatory mediators in the aqueous humor are associated with disease severity and the structural response to anti-VEGF therapy in BRVO. We specifically evaluated TNeuro (the thickness of the neurosensory retina) to distinguish intraretinal edema from subretinal fluid. This distinction is expected to provide additional insight into inflammatory retinal swelling independently of serous detachment. Given that inflammatory cytokines are thought to primarily affect vascular permeability within the neurosensory retina, TNeuro may more accurately reflect inflammatory retinal thickening than total CMT alone.

In the present study, significant improvements in BCVA, CMT, TNeuro, SRT, and aqueous flare values were observed four weeks following IRI. These functional and anatomical benefits are consistent with previously reported outcomes [1,19,20]. The underlying mechanism may be explained by the positive feedback loop proposed by Campochiaro et al. [21], wherein vascular occlusion induces retinal ischemia, triggering the release of VEGF. This elevation in VEGF levels exacerbates retinal non-perfusion by inducing inflammation-associated leukostasis. Mechanistically, the binding of VEGF and placental growth factor (PlGF) to VEGF receptor-1 (VEGFR-1) promotes leukocyte chemotaxis and subsequent inflammation. Concurrently, VEGF binding to VEGFR-2 upregulates inflammatory mediators, such as monocyte chemoattractant protein-1 (MCP-1) and intercellular adhesion molecule-1 (ICAM-1), via the NF-κB pathway. Thus, VEGF and PlGF facilitate both the recruitment and adhesion of leukocytes to the vascular endothelium, driving inflammatory responses. These processes likely disrupt the blood–aqueous barrier, leading to protein leakage from iridal vessels and a subsequent increase in aqueous flare. Our findings suggest that treatment with IRI inhibits these inflammatory cascades by suppressing VEGFR-1 and VEGFR-2 signaling, thereby reducing both aqueous flare and macular edema. Beyond these clinical improvements, we demonstrated that aqueous humor concentrations of VEGF, as well as the emerging inflammatory mediators Flt-3L and endocan-1, were markedly elevated in patients with BRVO compared with control subjects. These findings indicate that the inflammatory milieu of BRVO is not solely driven by VEGF, but rather involves a broader network of cytokines and endothelial-related factors.

Flt-3L is a type I transmembrane protein that can be released into the extracellular space as a soluble cytokine [22,23]. It is essential for hematopoietic stem cell expansion and lineage commitment, and it serves as a central modulator of dendritic cell development and activation [22,23]. In addition, Flt-3L has been shown to promote lymphocyte proliferation [24]. Elevated Flt-3L expression has been implicated in sustained immune activation and chronic inflammatory states affecting multiple organs, including the lung, central nervous system, and gastrointestinal tract [25,26]. Our findings suggest that similar mechanisms may operate in the retinal environment of BRVO. Specifically, aqueous humor Flt-3L levels showed significant associations with aqueous flare values and with concentrations of fractalkine, CXCL16, and endocan-1. Importantly, Flt-3L was also significantly correlated with TNeuro, highlighting a potential link between inflammatory signaling and neuroretinal structural changes. Collectively, these observations support the notion that Flt-3L, alongside VEGF, plays a substantial role in the inflammatory cascade underlying BRVO-related macular edema. We propose that Flt-3L may function as an immunological interface connecting innate and adaptive immune responses in BRVO. Oxidative stress, which is known to be enhanced in ischemic retinal conditions, may induce Flt-3L expression [27,28]. In turn, increased Flt-3L could promote the recruitment and activation of dendritic cells, thereby amplifying local inflammatory responses within the macular region. Interestingly, higher baseline aqueous Flt-3L levels were associated with greater reductions in CMT and TNeuro at four weeks after IRI, suggesting that elevated pre-treatment Flt-3L may reflect an inflammatory phenotype that is particularly responsive to therapy. Nonetheless, the precise molecular pathways through which Flt-3L influences BRVO progression and treatment responsiveness remain to be clarified in future studies.

Endocan-1, formerly known as endothelial cell-specific molecule-1, is a soluble dermatan sulfate proteoglycan expressed predominantly in endothelial tip cells during neovascular sprouting and is closely linked to angiogenic activity [29,30,31,32]. In this study, aqueous humor endocan-1 levels were significantly increased in BRVO patients. Previous studies have demonstrated that hypoxia-related signaling molecules, including VEGF and proinflammatory cytokines, induce endocan-1 expression [29,33,34,35]. Our data further suggest that endocan-1 is highly responsive to oxidative stress within the vascular endothelium [36]. Given the strong correlations observed between endocan-1, Flt-3L, CXCL16, and aqueous flare values, endocan-1 may serve as a sensitive indicator of oxidative endothelial injury. In BRVO, reactive oxygen species-mediated endothelial damage likely stimulates endocan-1 release, reflecting the extent of endothelial dysfunction and compromise of the blood–retinal barrier. This process may ultimately contribute to increased vascular permeability and the progression of macular edema.

Interestingly, aqueous humor levels of fractalkine and CXCL16 measured immediately prior to IRI in the BRVO group were not significantly higher than those measured at the time of cataract surgery in control subjects. This contrasts with our previous findings in patients with central retinal vein occlusion, in whom both chemokines were markedly elevated [16]. This discrepancy may be explained by the more extensive retinal ischemia typically observed in CRVO compared with BRVO. CXCL16 is a multifunctional chemokine with potent angiogenic properties and plays a key role in directing endothelial progenitor cell migration [37]. It has also been reported as a prognostic biomarker in a range of pathological conditions, including malignancies, infectious diseases, and systemic sclerosis [38,39,40]. In the current study, CXCL16 levels were significantly correlated with aqueous flare values in patients with BRVO, suggesting a relationship between intraocular inflammation and CXCL16-mediated signaling. These findings imply that CXCL16 may be induced during the early inflammatory phase of BRVO, potentially through VEGF-driven activation of infiltrating immune cells such as macrophages. The observed correlations among CXCL16, Flt-3L, and fractalkine further support a coordinated inflammatory network contributing to leukocyte recruitment and tissue remodeling. Although CXCL16 concentrations themselves were not significantly elevated in BRVO compared with controls, their close association with aqueous flare values and Flt-3L suggests potential clinical relevance. CXCL16 is uniquely upregulated by oxidized low-density lipoprotein and acts as a scavenger receptor for both oxidized lipoproteins and phosphatidylserine [41]. This property raises the possibility that lipid peroxidation, a hallmark of oxidative retinal injury, may activate CXCL16-dependent pathways during the acute phase of BRVO, thereby facilitating macrophage infiltration. Further experimental and clinical studies are required to delineate the precise role of CXCL16 in BRVO pathophysiology.

In addition, microglia may play a pivotal role in the pathogenesis of BRVO. Once activated, microglia produce various inflammatory cytokines, thereby exacerbating neuroinflammation [42,43,44]. Furthermore, microglial activation has been linked to hypoxia-induced VEGF expression, suggesting a pathophysiological link between vascular changes and neuroinflammatory processes [45,46,47]. Therefore, microglia may represent a key cellular mediator integrating ischemia, angiogenesis, and inflammation in this condition.

A comparison of the inflammatory profiles identified in the BRVO cases of this study with those previously reported for CRVO [16] is summarized in Table 5. Although both conditions exhibit elevated levels of VEGF, the increase is significantly more pronounced in CRVO than in BRVO. This finding indicates a shared pathway of ischemia-induced neovascularization, albeit with differing magnitudes of expression. Furthermore, both CRVO and BRVO demonstrate increased levels of Flt-3L. Notably, in BRVO, this marker appears to possess predictive value, suggesting a potential differential prognostic role for Flt-3L in assessing disease progression between the two types of occlusion. Similarly, Endocan-1 levels are consistently elevated in both conditions, pointing toward a common mechanism of endothelial activation. Regarding clinical parameters, CRVO displays only a weak-to-moderate correlation with aqueous flare, suggesting a more generalized ocular involvement. Conversely, BRVO exhibits a strong correlation with flare, which may reflect “segmental inflammation” localized specifically to the affected retinal area. Thus, this comparative analysis of inflammatory markers and clinical parameters reveals distinct profiles for CRVO and BRVO, suggesting nuanced differences in their underlying pathophysiological mechanisms.

Several limitations of this study should be acknowledged. The sample size was relatively small, and all participants were recruited from a single institution, which may limit the external validity of the results. Additionally, the observational nature of the study introduces the possibility of selection bias. Moreover, although differences between macular BRVO and major BRVO potentially influence the inflammatory response, no significant differences were observed between subgroups, likely due to the small sample size. Given the limited statistical power of the subgroup analysis, further large-scale research is warranted. Finally, the follow-up period was relatively short, precluding evaluation of long-term retinal structural changes. Larger, multicenter prospective studies with extended follow-up durations are warranted to confirm and expand upon these findings.

## 5. Conclusions

**Figure 4:**
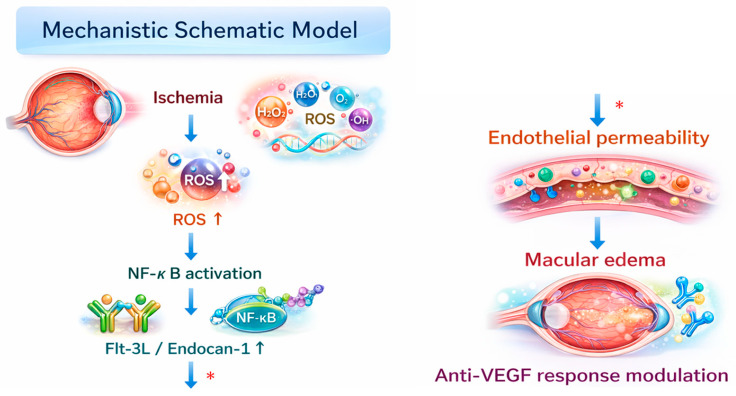
Proposed mechanistic schematic model of oxidative stress–inflammatory signaling in branch retinal vein occlusion (BRVO). Red asterisks denote hypothesized mechanisms based on the current results.

In conclusion, aqueous humor concentrations of VEGF, together with the emerging inflammatory mediators Flt-3L and endocan-1, were significantly elevated in patients with BRVO compared with control subjects. Significant interrelationships were also identified among Flt-3L, CXCL-16, and endocan-1, as well as between these factors and aqueous flare values, indicating a close link with intraocular inflammatory and oxidative activity. Importantly, in patients with BRVO, baseline aqueous Flt-3L levels were significantly associated with subsequent changes in macular edema, including alterations in CMT and TNeuro. These findings suggest that these novel inflammatory mediators are integral components of a complex oxidative–inflammatory network initiated by retinal ischemia, in which excessive reactive oxygen species generation may act as an upstream trigger. A schematic representation summarizing the proposed oxidative stress–inflammatory cascade underlying BRVO-associated macular edema is presented in Figure 4. From an antioxidant perspective, dysregulation of redox homeostasis in the retinal microenvironment may promote endothelial dysfunction, immune cell activation, and blood–retinal barrier breakdown through Flt-3L- and endocan-1-related pathways.

Accordingly, therapeutic strategies aimed at restoring redox balance—either through direct antioxidant approaches or by targeting redox-sensitive inflammatory mediators—may represent a promising adjunct or alternative to conventional anti-VEGF therapy. Such antioxidant-oriented interventions may be particularly beneficial for patients with BRVO who exhibit persistent inflammation and macular edema despite standard treatment, and warrant further investigation.

Retinal ischemia induced by BRVO leads to excessive generation of reactive oxygen species (ROS), which acts as an upstream trigger for oxidative stress-mediated inflammatory signaling. Increased ROS activates redox-sensitive transcription pathways, including nuclear factor-κB (NF-κB), resulting in the upregulation of inflammatory mediators such as Flt-3 ligand (Flt-3L) and endocan-1. These mediators contribute to endothelial dysfunction and increased vascular permeability, leading to breakdown of the blood–retinal barrier and the development of macular edema. This oxidative–inflammatory cascade may also influence the therapeutic response to anti-vascular endothelial growth factor (anti-VEGF) treatment. The schematic summarizes the proposed interactions between ischemia, oxidative stress, inflammatory mediators, and vascular permeability in the pathogenesis of BRVO-associated macular edema.

## Figures and Tables

**Table 1:** Aqueous Humor Factors in the Control and BRVO Groups.

	Control	BRVO	*p* Value
VEGF (pg/mL)	60.4 ± 22.4	155 ± 128	<0.001
Flt-3L (pg/mL)	0.99 ± 0.25	1.45 ± 0.48	<0.001
Fractalkine (pg/mL)	19.1 ± 2.14	20.3 ± 7.0	0.560
CXCL-16 (pg/mL)	232 ± 136	263 ± 145	0.579
Endocan-1 (pg/mL)	276 ± 85.9	632 ± 452	<0.001

BRVO = branch retinal vein occlusion; VEGF = vascular endothelial growth factor; Flt-3L= FMS-related tyrosine kinase 3 ligand; CXCL16 = CXC chemokine ligand 16.

**Table 2:** Correlations Between Aqueous Humor Factors and the OCT Parameters.

	VEGF	Flt-3L	Fractalkine	CXCL-16	Endocan-1
Variable	*r*	*r*	*r*	*r*	*r*
	*p* Value	*p* Value	*p* Value	*p* Value	*p* Value
CMT	0.41	0.33	0.32	0.21	0.27
	0.025	0.075	0.082	0.264	0.146
TNeuro	0.22	0.39	0.32	0.33	0.32
	0.241	0.031	0.083	0.071	0.090
SRT	−0.15	0.15	0.08	0.24	0.15
	0.445	0.423	0.677	0.205	0.431
Aqueous flare	0.54	0.46	0.11	0.37	0.59
	0.002	0.011	0.563	0.046	<0.001

OCT = optical coherence tomography; VEGF = vascular endothelial growth factor; Flt-3L = FMS-related tyrosine kinase 3 ligand; CXCL16 = CXC chemokine ligand 16; CMT = central retinal thickness; TNeuro = thickness of the neurosensory retina; SRT = serous retinal thickness; *r* = correlation coefficient.

**Table 3:** Correlation Matrix For Aqueous Humor Factors.

	VEGF	Flt-3L	Fractalkine	CXCL-16	Endocan-1
Variable	*r*	*r*	*r*	*r*	*r*
	*p* Value	*p* Value	*p* Value	*p* Value	*p* Value
VEGF		0.31	0.22	0.18	0.61
		0.098	0.248	0.346	<0.001
Flt-3L			0.38	0.54	0.60
			0.037	0.002	<0.001
Fractalkine				−0.39	−0.17
				0.034	0.364
CXCL-16					0.75
					<0.001

VEGF = vascular endothelial growth factor; Flt-3L = FMS-related tyrosine kinase 3 ligand; CXCL16 = CXC chemokine ligand 16; *r* = correlation coefficient.

**Table 4:** Correlations between baseline aqueous humor levels of growth factors and inflammatory mediators and changes in clinical factors.

	Improvement of BCVA	Change in CMT	Change in TNeuro	Change in Aqueous Flare
Variable	*r*, *p* Value	*r*, *p* Value	*r*, *p* Value	*r*, *p* Value
VEGF (pg/mL)	0.16, 0.398	0.16, 0.409	0.08, 0.665	0.17, 0.377
Flt-3L (pg/mL)	0.25, 0.190	0.39, 0.036	0.39, 0.032	0.07, 0.703
Fractalkine (pg/mL)	0.12, 0.545	0.31, 0.092	0.24, 0.197	0.15, 0.418
CXCL-16 (pg/mL)	0.07, 0.709	0.06, 0.771	0.17, 0.375	0.06, 0.754
Endocan-1 (pg/mL)	0.06, 0.770	0.22, 0.243	0.25, 0.183	0.12, 0.522

BCVA = best-corrected visual acuity; CMT = central retinal thickness; TNeuro = thickness of the neurosensory retina; VEGF = vascular endothelial growth factor; Flt-3L= FMS-related tyrosine kinase 3 ligand; CXCL16 = CXC chemokine ligand 16; *r* = correlation coefficient.

**Table 5:** Differences in Local Inflammation Control Between CRVO and BRVO.

Marker	CRVO [16]	BRVO [Current]	Interpretation
VEGF	↑↑	↑	Shared pathway
Flt-3L	↑	↑ (predictive)	Possible differential prognostic role
Endocan-1	↑	↑	Endothelial activation
Flare correlation	weak/moderate	strong	Segmental inflammation?

CRVO = central retinal vein occlusion; BRVO = branch retinal vein occlusion; VEGF = vascular endothelial growth factor; Flt-3L = FMS-related tyrosine kinase 3 ligand. ↑ indicates increased levels; ↑↑ indicates markedly increased levels.

## Data Availability

The original contributions presented in this study are included in the article. Further inquiries can be directed to the corresponding author.

## Abbreviations

The following abbreviations are used in this manuscript:

BCVA	best-corrected visual acuity
BRVO	branch retinal vein occlusion
CMT	central macular thickness
CRVO	central retinal vein occlusion
FA	fluorescein angiography
Flt-3L	FMS-related tyrosine kinase 3 ligand
IRI	intravitreal ranibizumab injection
LogMAR	logarithm of the minimum angle of resolution
OCT	optical coherence tomography
Ox-LDL	oxidized low-density lipoprotein
ROS	reactive oxygen species
SRT	subfoveal serous retinal thickness
SD-OCT	spectral-domain optical coherence tomography
TNeuro	neurosensory retinal thickness
VEGF	vascular endothelial growth factor
